# Evaluation of Two Anorganic Bovine Xenogenous Grafts in Bone Healing of Critical Defect in Rats Calvaria

**DOI:** 10.1590/0103-6440202406119

**Published:** 2024-10-25

**Authors:** Valdir Gouveia Garcia, Gizelli de Souza Dall´Agnol, Christian Cézane Cardoso Campista, Luiz Lordêlo Bury, Edilson Ervolino, Mariéllen Longo, Gabriel Mulinari-Santos, Liran Levin, Letícia Helena Theodoro

**Affiliations:** 1 Latin American Institute of Dental Research and Teaching, Ilapeo College, Curitiba, Paraná, Brazil.; 2 Graduate Program in Dentistry, Faculty of Dentistry of Barretos, Barretos, São Paulo, Brazil.; 3 Department of Basic Sciences, São Paulo State University (UNESP), School of Dentistry, Araçatuba, São Paulo, Brazil.; 4 Department of Diagnosis and Surgery, São Paulo State University (UNESP), School of Dentistry, Araçatuba, São Paulo, Brazil.; 5 Faculty of Medicine and Dentistry, University of Alberta, Canada.

**Keywords:** Bone regeneration, bone substitutes, wound healing, rats, xenograft

## Abstract

The purpose of this study was to provide an evaluation of two different xenogeneic bone substitutes in bone healing of critical-sized bone defects (Ø =5mm) created in rats calvaria. Thirty animals were randomized into 3 groups with one of the following treatments. In the control group (n=10), the defects were filled with blood clots; BO group (n=10), the defects were filled with bovine medullary bone substitute (Bio-Oss®); BF group (n=10), the defects were filled with bovine cortical bone substitute (Bonefill®). All defects were covered with an absorbable membrane. Five animals from each group were euthanized at 30 and 45 days, subsequently histomorphometrical and immunohistochemical analyses were performed. The histomorphometry was used to measure the percentage of new bone formation in the total area of the defect while the immunohistochemistry evaluated the expression of bone immunomarkers for bone morphogenetic protein 2/4 (BMP2/4), osteocalcin (OCN) and tartrate-resistant acid phosphatase (TRAP). Data was statistically analyzed with a 5% significance level. The results demonstrated that the BO group showed greater bone formation compared to the BF group at 30 days (P<0.05). However, there was no statistically significant difference between the control and BO groups at 30 days (P>0.05). The expression of BMP2/4 and OCN were higher in the BO group at 45 days compared to the BF at 30 and 45 days respectively (P<0.05). In conclusion, even with the higher expression of proteins related to bone formation, there was no difference in new bone formation at 45 days when both anorganic bovine xenogenous grafts were evaluated.

## Resumo

O objetivo deste estudo foi realizar uma avaliação comparativa de dois diferentes substitutos ósseos xenogênicos na consolidação óssea de defeitos ósseos de tamanho crítico (Ø = 5 mm) criados em calvária de ratos. Trinta animais foram randomizados em 3 grupos: no grupo controle (n=10), os defeitos foram preenchidos com coágulos sanguíneos; no grupo BO (n=10), os defeitos foram preenchidos com substituto ósseo medular bovino (Bio-Oss®) e no grupo BF (n=10), os defeitos foram preenchidos com substituto ósseo cortical bovino (Bonefill®). Todos os defeitos foram cobertos com membrana absorvível. Cinco animais de cada grupo foram eutanasiados aos 30 e 45 dias de pós-operatório, logo após foram realizadas análises histomorfométricas e imuno-histoquímicas. A histomorfometria foi utilizada para mensurar a porcentagem de neoformação óssea na área total do defeito original enquanto a imunohistoquímica avaliou a expressão de imunomarcadores ósseos para proteína morfogenética óssea 2/4 (BMP2/4), osteocalcina (OCN) e fosfatase ácida resistente ao tartarato (TRAP). Todos os dados foram analisados estatisticamente com nível de significância de 5%. Os resultados demonstraram que o grupo BO apresentou maior formação óssea em comparação ao grupo BF aos 30 dias (P<0,05). No entanto, não houve diferença estatística entre os grupos controle e BO aos 30 dias (P>0,05). As expressões de OCN e BMP2/4 foram maiores no grupo BO aos 45 dias em comparação ao BF aos 30 e 45 dias respectivamente (P<0,05). Concluiu-se que mesmo com a diferença da alta expressão de proteínas relacionadas a formação óssea, ambos os biomateriais não demonstraram diferenças na indução de formação óssea após 45 dias pós-operatório.

**Figure:**
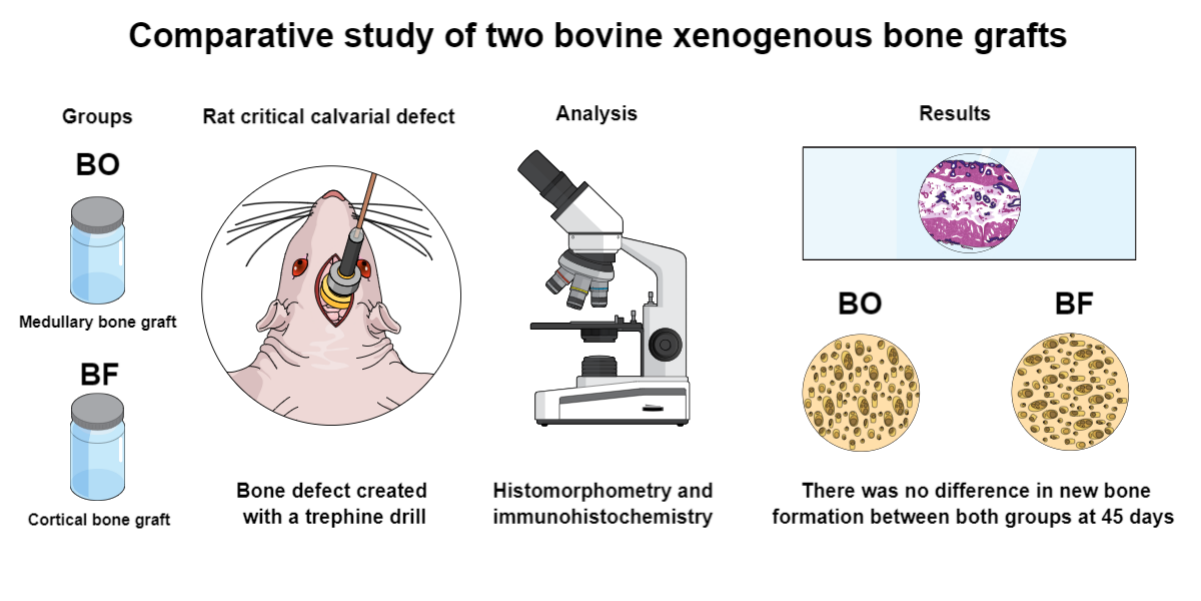

## Introduction

Bone substitutes play a significant role in implant dentistry, providing crucial support for bone healing, reconstruction, and augmentation ^1^. In cases of bone loss or damage, the use of bone graft substitutes is essential to ensure proper support as a scaffold and to stimulate the new bone formation ^1^. Bone graft substitutes encompass a range of substances used to fill bone defects and induce new bone formation ^2^. Biomaterials can be obtained from various sources, including the patient's own body called autografts, from humans or animals termed allografts and xenografts, or from synthetic compounds named alloplastic ^2^. Each of these alternatives has specific characteristics, making them suitable for different clinical scenarios ^3^. Various biomaterials have been proposed as alternatives to autogenous bone grafts since they require additional surgery and possible donor-site morbidity ^1^. Thus, widely used alternatives include anorganic xenogenous bovine bones ^2^.

Bio-Oss® (BO) is considered one of the most effective and widely used anorganic bovine xenogenous grafts ^4^. This biomaterial is commonly used as a reference in studies comparing other bone substitutes when autografts are not used ^5^. The efficiency and compatibility of BO have been extensively studied in surgical procedures, such as sinus floor elevation and implant placement ^4^ ^,^ ^6^. BO is a medullary bovine bone substitute designed to mimic the properties of natural human bone, enabling integration and bone regeneration ^7^. BO particles are meticulously crafted through a precise heating process at 300°C, and then undergo a thorough treatment with sodium hydroxide^8^. BO particles stimulate the deposition of calcium and phosphate, promoting the formation of hydroxyapatite, a key inorganic component of the bone matrix ^7^. BO has a favorable environment for bone regeneration with interconnected porosity, allowing the ingrowth of blood vessels and cellular infiltration ^9^. Despite BO being frequently recommended, there is a vast array of other bone substitutes available, and their possible prominent worth needs to be explored.

Bonefill® (BF) is an anorganic bovine xenogenous graft derived from the cortically bovine femurs ^10^. BF is manufactured through a sequential bath process designed to effectively solubilize the organic component from its particles ^8^. It has been widely discussed in the literature with promising results ^10^ ^,^ ^11^. Different studies including histomorphometry ^4^ and physicochemical analysis^8^ showed its composition and potential to serve as an alternative bone substitute. The use of BF has been investigated in comparison to other bone substitutes such as BO ^4^ ^,^ ^8^ ^,^ ^12^. Previous studies have analyzed BF in various preclinical applications, including bone healing in rabbits calvaria ^4^, calvaria critical-size defects in rats ^12^ and alveolar bone healing in rats ^11^. The results demonstrated that BF exhibits favorable osteoconductive properties, mainly providing a scaffold for osteogenesis ^4^. Beside animal studies, clinical reports also demonstrated convincing findings of the BF in the form of blocks for maxilla reconstruction ^13^ or granule particles for sinus augmentation ^14^. Moreover, studies assessing the long-term stability of BF have reported that this bone substitute maintains its structural integrity over a long time ^12^ and under dissolution conditions ^8^.

According to the available literature, there is a need for further investigations evaluating these bone biomaterial substitutes in different scenarios ^4^ ^,^ ^12^. Although there is wide use of BO, some findings indicate superior outcomes when using BO in comparison with other bone substitutes ^5^ ^,^ ^15^ while others have not reported additional benefits ^4^ ^,^ ^12^. Understanding the intricate nuances of bone biomaterial substitutes is crucial for clinicians and researchers. It supports them in making informed decisions regarding the most suitable preferences for patients with different bone defects and treatment requirements. Therefore, this article aimed to evaluate and compare through histomorphometry and immunohistochemical analysis the characteristics of two anorganic bovine xenogenous grafts (BO and BF) by examining their regenerative properties using a standardized model of bone healing in critical-sized defects in rats calvaria.

## Materials and Methods

### Animals

The study was approved by the Ethics Committee on Animal Experimentation of the Faculty of Dentistry (process 1200-03/2011), in accordance with the current norms adopted by the Brazilian College of Animal Experimentation. Additionally, all steps of the experiment carefully followed the Arrive Guidelines 2.0 ^16^

### Sample size

A total of thirty male rats (Rattus norvegicus Albinus, Wistar), 3 to 4 months of age, weighing approximately 250 to 300g, kept in an environment with stable temperature (22 ± 2°C), with water and food ad libitum, were used for this study. The sample size was estimated according to the previous literature, practice, and performance of a pilot study. It was assumed, as established by Grossi-Oliveira et al. ^12^, that four animals were adequate to ensure statistical significance for histological analysis. Based on this, and to compensate for possible dropouts, while minimizing the number of animals, the sample size for this parameter was n=5. Previous experience of our research group using the same rodent model ^17^ determined that n = 10 per group was sufficient to reject the null hypothesis in the other analyses. The estimation for each outcome measure was performed to achieve a 0.8 power and 0.05 alpha error.

### Study design

Simple randomization of the animals (1:1 allocation ratio) into groups was performed through a computer-generated random number table by a blinded external member of the study. Numerals from 1 to 30 were labelled on the tails of the rats with a drawing pen. The number order was uploaded to the software (Minitab® 17 Minitab Inc., State College, PA, USA). The animals were randomly assigned into three experimental groups of ten animals per group. Next, each animal received one of the following treatments: Control group (n=10), defects were filled with blood clot; BO group (n=10), the defects were filled with inorganic hydroxyapatite extracted from the medullary portion of the tibia bovine (particles of 0.25-1 mm in diameter; Bio-Oss®, Geistlich Pharma AG, Switzerland); BF group (n=10), the defects were filled with anorganic bone matrix extracted from cortical bovine femur bone (particles of 0.6-1.5 mm in diameter, Bonefill®, Bionnovation Produtos Biomédicos S.A., Bauru, SP, Brazil). Both bone biomaterial substitutes were inserted carefully into the bone defects until complete filling, without excessive condensation. All defects were covered with a collagen membrane of demineralized bovine cortical bone (GenDerm®, Baumer, SP, Brazil). Both bone biomaterial substitutes were hydrated with saline solution (0.9%) before implantation in the bone defect. Soft tissue sutures were performed with resorbable sutures (Vicryl 4-0, Ethicon, Johnson & Johnson, Somerville, NJ, USA). All evaluations were performed following calibration and blinding examination.

### Surgery

**Figure 1:**
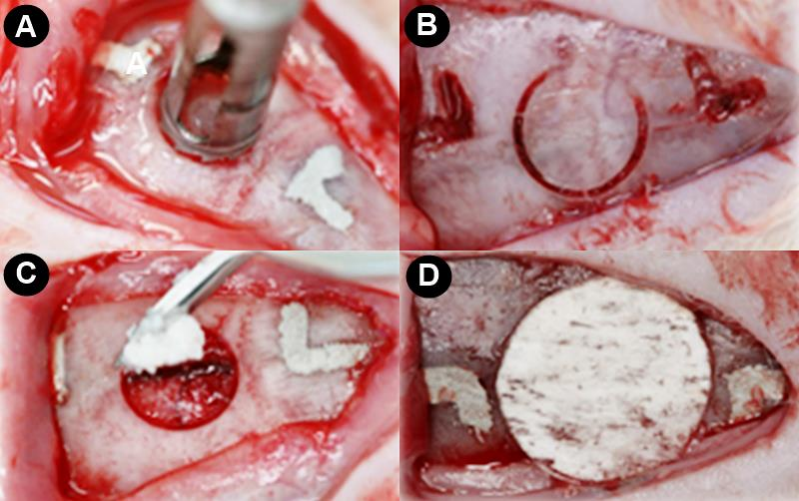
Creation of a critical-size bone defect in rats calvaria. A) Creation of a full-thickness flap with a semilunar incision and initiation of osteotomy using trephine drill; B) Osteotomy performed to remove the bone fragment; C) Beginning of filling the bone defect with biomaterial; D) Bovine bone cortical membrane installed over the biomaterial.

All animals received general anesthesia with 80 mg/kg of ketamine hydrochloride (Cetamin, Syntec do Brasil Ltd., Cotia, SP, Brazil) and 60 mg/kg of Xylazine Hydrochloride (Xilazin, Syntec do Brasil Ltd., Cotia, SP, Brazil) administered intramuscularly to the right leg of the animal. When supplementation was needed for anesthesia, a dose equivalent to 50% of the initial dose was applied. After asepsis and trichotomy of the calvaria, a semilunar incision was performed, and a full-thickness flap was raised. Next, a single bone defect of critical size was created (Ø=5mm) in the central portion of the calvaria, performed with a trephine drill (Dentoflex, System of Implantes, São Paulo, SP, Brazil) mounted on an implant engine with reduced speed and under abundant irrigation with sterilize saline solution (0.9%). It was carried out twice ¨L¨-shaped markings from the edge of the defect created using a drill spherical no. 2 mm posterior and 2 mm anterior to the margin of the defect, and its major axis followed the median sagittal plane of the animal and served to guide the histological processing and evaluation. The technique for creating the bone defects followed a previously described and validated protocol ^18^ as demonstrated in the Figure 1.

### Euthanasia and histology process

Five animals from each group were euthanized at 30 and 45 days after surgery. The calvaria of these animals, containing the created bone defects, were removed and preserved for 48 hours in a 4% formaldehyde solution. They were then washed in running water for 24 hours, and the demineralization process started, carried out in a solution of Acid 16% ethylenediaminetetraacetic (EDTA) in the proportion of 250 mg per 1750 ml of distilled water. After the parts were washed and embedded in paraffin blocks. Six serial sections were made 5 µm thick from the center of the bone defect. Half of these sections were stained by the Hematoxylin and Eosin (HE) technique and served for the histomorphometry analysis, while the others underwent processing for immunohistochemical analysis.

### Histomorphometry analysis

The histomorphometry analysis was performed using a computer image evaluation system, ImageLab 2000 software (Diracon Bio Informática Ltda., Vargem Grande do Sul, SP, Brazil), by a single examiner, calibrated and blinded to the periods and treatments. The technique for histomorphometry analysis followed previous established methods ^17^.

Histological sections were selected from the central area of each specimen's surgical defect in a sagittal direction. Each section was captured using a digital camera coupled to an optical microscope and saved on a computer. In each image, a delimitation of the analyzed area was performed, corresponding to the region of the calvaria where the defect was created, defined as the total area. This area was first determined by the identification of the external and internal surfaces of the original calvaria on the right and left margins of the surgical defect. These surfaces are related to drawn lines following their respective curvatures. Considering the total length of the histological specimen, 2 mm were measured from the right and left extremities of the specimen, towards the center of the defect, to identify the margins of the surgical defect.

The area of new bone formation occupied over the remnants of the implanted bone biomaterial substitutes, BO and BF, was delineated within the limits of the total area. The new bone formation of the respective specimens was evaluated three times by the same examiner and on different days. The three measurements obtained were statistically analyzed and a significant level was set at 5% using the Kappa test. Mean values were ascertained and statistically compared. Digital images were created with a combination of three images, because of the impossibility of capturing the entire defect in only one image due to the magnification used. The image was created using Adobe Photoshop® software (Adobe, San Jose, CA) concerning anatomical structures such as blood vessels and bone trabeculae in each of the histological sections.

### Immunohistochemical analysis

For immunohistochemical reactions, the slides were treated by indirect immunoperoxidase technique employing the primary polyclonal antibodies to bone morphogenetic protein 2/4 (1:100, anti-BMP2/4, sc137087, Santa Cruz Biotechnology, Santa Cruz, CA), osteocalcin (1:100, anti-OCN, sc365797, Santa Cruz Biotechnology, Santa Cruz, CA) and tartrate-resistant acid phosphatase (1:100, anti-TRAP, sc376875, Santa Cruz Biotechnology, Santa Cruz, CA). The primary antibodies were diluted in bovine serum albumin (BSA) with diluent (DAKO - Carpinteria, CA, USA) and normal serum (3%, Sigma, CA, USA).

Initially, the histological sections were deparaffinized at 56 °C for 30 min, and a second cycle of deparaffinization started with xylol baths, followed by rehydration in decreasing solutions of alcohols, and finally washed in successive baths in sodium phosphate buffer (SPB). After that, the slides were placed in a solution containing 198 ml of distilled water with 2 ml of 100X citrate buffer at 95 °C for 5 min for antigen retrieval.

The histological sections were treated for blockade of the endogenous peroxidase employing 3% hydrogen peroxide in SPB for 1 h and then washed again with SPB. Endogenous biotin blockade was performed with a solution containing SBP and skimmed milk powder 3% for 1 h. Blocking of non-specific sites was also performed with a solution of bovine serum albumin (BSA) overnight. Thereafter, the sections were incubated with the above-mentioned primary antibodies at room temperature for 18-24 hours and washed with SPB. A second incubation was performed using a universal biotinylated secondary antibody (Anti-Goat made in Horse, DAKO-Carpinteria, CA, USA) for 2 h at room temperature, followed by a wash with SPB. A third incubation was performed with a solution containing streptavidin conjugated to peroxidase (DAKO - Carpinteria, CA, USA) at room temperature for 2 h.

Immunoperoxidase reaction was performed with buffer (DAB-Substrate, DAKO - Carpinteria, CA, USA) and diaminobenzidine (DAB-Chromogen, DAKO - Carpinteria, CA, USA) for 5 min for BMP 2/4 and OCN, 60 s for TRAP at room temperature. Finally, histological sections were washed several times in SPB and counterstained for 15 seconds with hematoxylin. All immunoperoxidase reactions were accompanied by a negative control when primary antibodies were omitted.

Immunohistochemical analysis followed a previously establish method ^18^. Thus, immunolabelling located at both margins and the center of the defect was analyzed at 400X magnification by light microscopy. The expression of BMP 2/4 and OCN were measured semi-quantitatively using scores from 1 to 4 assigned as 1= absent, 2=mild, 3=moderate and 4=intense. TRAP-positive cells were counted, and the results were expressed in units. In order to be considered TRAP-positive cells, mature osteoclasts should contain three or more nuclei.

### Statistical analysis

Statistical analysis of the histometric and immunohistochemical data was performed by GraphPad Prism 9 software (GraphPad Software; La Jolla; CA; USA). The hypothesis that there was no statistically significant difference among the different groups and periods was tested. The normality of the data was evaluated by the Shapiro-Wilk test. A parametric normal distribution of the data for new bone formation and TRAP immunolabelling was observed. Therefore, their statistical test was performed by parametric analysis of variance ANOVA with Tukey complementation at p < 0.05. Otherwise, BMP2/4 and OCN obtained a nonparametric distribution, thus it was assumed Kruskal Wallis Analysis of Variance; Student Newman-Keuls post-test at p<0.05.

## Results

**Figure 2:**
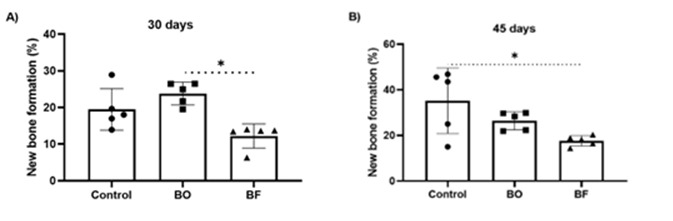
Figure 2. Scatter plot graphs with columns representing the histomorphometry analysis for all groups at 30 days (A) and 45 days (B). Symbols: *, a statistically significant difference in relation to the BF group. Statistical tests: ANOVA of Variance; Tukey post-test; p<0.05.

The histomorphometry showed that the new bone formation at 30 days was statistically higher in the BO group than in the BF group (24.8% ± 2.1 vs 13.6± 1.8; p=0.0071). The Control group presented a percentage of 19.9%±2.8 (Figure 2A).

The new bone formation at 45 days was statistically greater in the Control group compared to the BF group (37.7± 1.7 vs 18.9±1.6; P=0.0486). The BO group presented a percentage of 27.5±1.5 of new bone formation at 45 days (Figure 2B).

**Figure 3:**
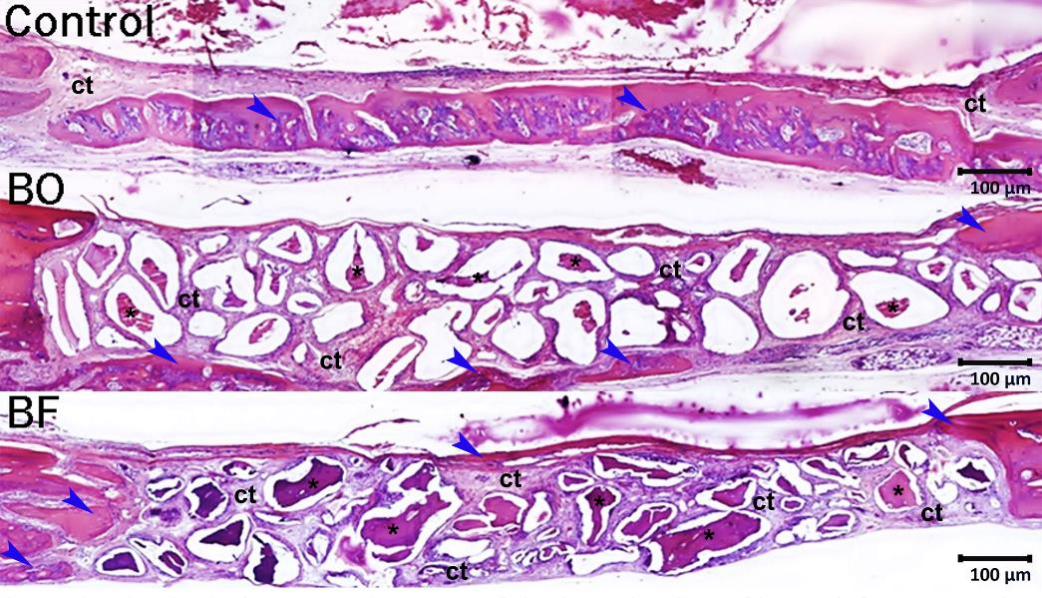
Histological panoramic aspect of the bone healing of bone defects at 45 days from the Control, BO, and BF groups respectively. Histological images reveal the presence of newly formed bone within the borders of the surgical defects in the Control group. Furthermore, a delicate layer of newly formed bone is observed on the surface of the remaining bone grafts in the BO and BF groups. Abbreviations and symbols: asterisks, bone graft remnants; ct, connective tissue; blue arrows, newly formed bone tissue. Original magnifications: 50x. Scale bars: 100 µm.

As seen in Figure 3, a narrow band of newly formed bone tissue along the edges of the surgical wound was observed. The patterns of new bone in the BO and BF groups were similar. However, the Control group exhibited a greater amount of bone compared to the BF group at 45 days. All bone defects in each group were filled with thin layer dense connective tissue. This connective tissue layer had a thin amount of collagen fibers aligned parallel to the wound surface, along with a small number of inflammatory cells, fibroblasts, and blood vessels.

The immunohistochemical technique used to detect BMP2/4, OCN and TRAP showed high specificity, which was confirmed by the absence of total labelling in the negative control of the reaction. The immunoreactive cells showed a dark brown coloration confined exclusively to the cytoplasm, in the case of TRAP, and confined to the cytoplasm and, to a lesser extent, to the extracellular matrix, in the case of BMP2/4 and OCN.

**Figure 4:**
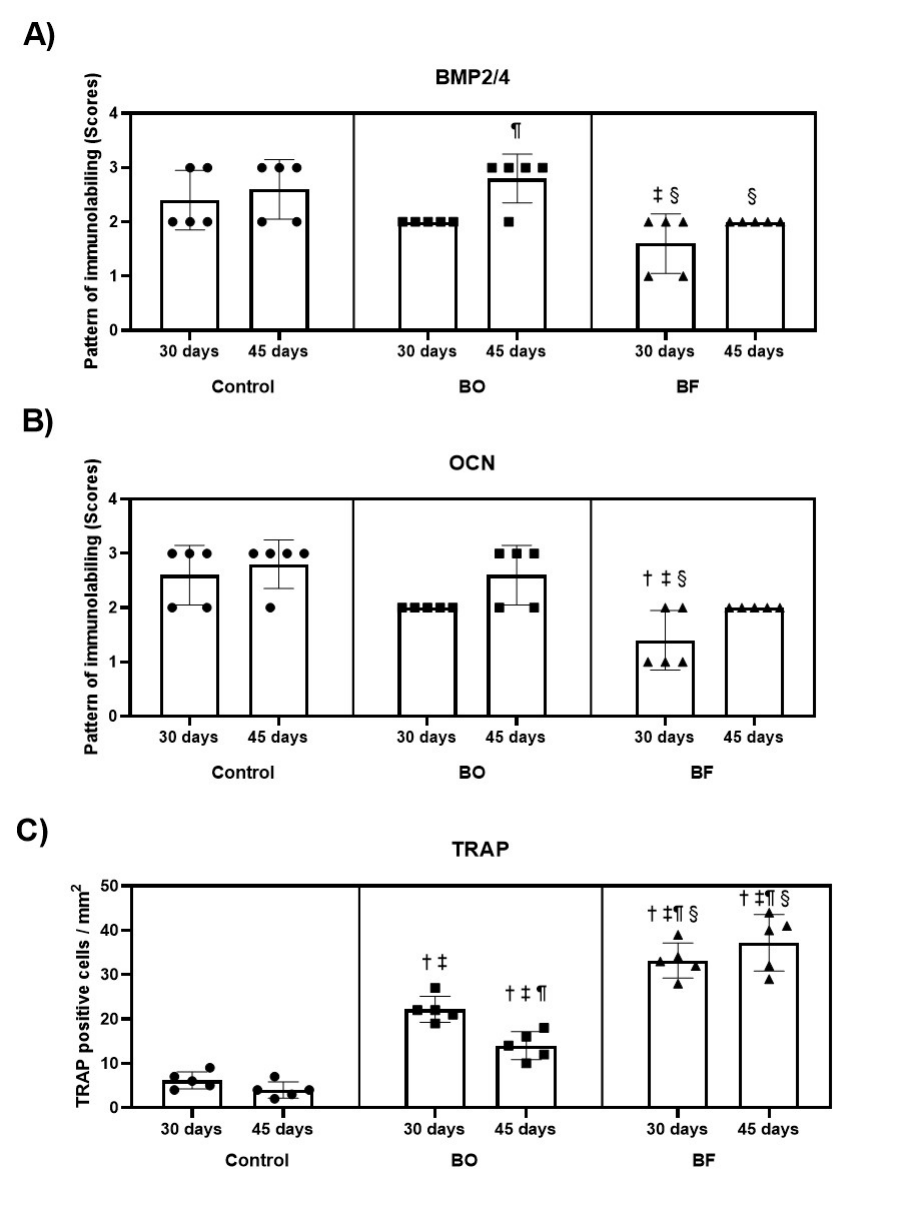
Scatter plot graphs with columns representing the immunohistochemical results for BMP2/4 (A), OCN (B) and TRAP (C). Statistical analysis of the immunostaining pattern by scores for BMP2/4, OCN and TRAP-positive cells per mm^2^ in bone defects in Control, BO and BF at 30 and 45 days postoperatively. Symbols: †, a statistically significant difference in relation to control group at 30 postoperative days; ‡, a statistically significant difference in relation to control group at 45 postoperative days; ¶, a statistically significant difference in relation to the BO group at 30 postoperative days; §, a statistically significant difference in relation to the BO group at 45 postoperative days. Statistical tests for BMP2/4 and OCN: Kruskal Wallis Analysis of Variance; Student Newman-Keuls post-test; p<0.05. Statistical test for TRAP: ANOVA of Variance; Tukey post-test; p<0.05.

Analysis of the immunostaining of the BMP2/4 and the OCN revealed that the medians of specimens from the Control and BO groups demonstrated the similarity of results of BMP 2/4 and OCN in both evaluation periods (Figure 4A and 4B). During the 30 and 45-day period, the BO group showed slightly more BMP2/4 immunostaining compared to the BF group. At 30 days, the BF group had lower levels of OCN expression compared to BO group, but there was no significant difference observed at 45 days.

In the analysis of the TRAP immunostaining, the specimens from the BF group showed the highest means of immunostaining positive cells in both periods (33.2 ± 4.0; 37.2 ± 6.4; Figure 4C); when compared to the BO and Control groups during the same periods.

## Discussion

Various bone biomaterials have been proposed as alternatives to autogenous bone grafts in oral surgeries ^1^. Some of these alternatives include xenogenous biomaterials such as the products tested in this study, since these biomaterials aim to provide a suitable scaffold for bone regeneration by their osteoconductive properties ^19^. The choice of a bone substitute depends on several factors, including the specific clinical application, patient characteristics, and the surgeon preference ^20^. It is important to note that biocompatibility and effectiveness can vary depending on consolidation with progressive apposition of new bone followed by resorption and replacement by bone ^21^. Additionally, it is necessary to understand and evaluate the osteoconductive and osteoinductive properties as a guidance to select the most suitable bone substitute ^20^. The main results from the histomorphometry and immunological analysis demonstrated that BO and BF presented equivalent quantitative amount of new bone formation at the final evaluated period. However, BO has revealed greater expression of bone proteins of OCN at 30 days and BMP2/4 at 45 days, and lower number of TRAP-positive cells in both periods. On the other hand, the BF group exhibited a delay in bone formation compared to the BO group within a 30-day period.

The results obtained in the present study showed that immunostaining for BMP2/4 and OCN in the BO group was equivalent with those of the control group in both evaluation periods. This finding highlights the importance of neo-angiogenesis to promote platelet adhesion and stimulates mesenchymal cells for bone formation through the presence of blood. Bone cell precursors from angiogenesis are crucial factors for bone healing and its continuous remodeling ^22^. In the comparative analysis of the biomaterials, it was observed that both products demonstrated capacity for bone neoformation, however, BO demonstrated immunostaining of BMP2/4 and OCN slightly higher than that of BF group, standing out in the period of 45 days. Then, it was observed that BO led to a positive induction of proteins that signaled the process of osteoblastic differentiation and maturation. The difference can be directly attributed to the temperature process of the bovine bone denaturation. BO undergoes high-temperature preparation at 300ºC, while BF is crafted at a lower temperature ^19^. This can result in a higher purification of BO and consequently lead to improved immune responses of bone proteins ^19^. The findings also showed that BF exhibited prolonged osteoclast activity, characterized by the higher number of TRAP-positive cells in both evaluation periods. These outcomes suggest that BF is capable to promote reabsorption and bone formation due to its osteoconductive properties.

An earlier study comparing BF and BO in rabbits through histometric and immunohistochemical analysis found that BF and BO both showed similar bone formation and integration ^4^. There were no significant differences observed in bone density and inflammation between the two products ^4^. However, it was concluded that further research and clinical trials were still necessary to fully evaluate the long-term compatibility and performance of these materials ^4^. Another clinical study revealed effective outcomes of implant osseointegration after implant placement and simultaneous sinus augmentation with BF ^14^. Furthermore, a prior animal study in rats found no significant differences in bone volume and density of BF associated or not with active oxygen-based oral gel ^11^. Regarding biocompatibility, both have previously demonstrated safe biocompatibility, meaning they are well-tolerated and capable of promoting bone regeneration ^4^ ^,^ ^14^ . In the applications aspect, BF has been effective in challenging bone healing situations, including rats submitted to experimental alcoholism ^23^, regenerative treatment in human mandibular class II furcation defects ^24^ and sinus augmentation ^14^.

The comparable features of the two tested materials might also be attributed to the porosity present in both materials as mentioned before ^4^. Both biomaterials exhibit an external surface of microporous ^8^ ^,^ ^19^, which enhances the superficial area for angiogenesis and bone regeneration. The two grafts have different particle sizes, which are important factors for cell adherence and cytokine release^4^ ^,^ ^8^. Interestingly, the BO graft has nanopores of 15-nm hydroxyapatite crystallites, and BO particles are smaller than BF granules. This might allow a larger surface area for bone cell adherence of endogenous proteins related to bone formation ^8^. Additionally, the manufacturing process significantly influences the osteoconductive features of these biomaterials. BO is manufactured by heating at 300 °C followed by a treatment with sodium hydroxide, while BF receives only a sequential bath to solubilize the organic part ^8^. The heating treatment results in a large surface area and a polyhydric format, consequently it can enhance bone formation by BO ^8^. BO has shown a higher dissolution ratio in acid conditions, which contributes to its resorption and bone formation ^8^. Moreover, BO was found to be free of organic parts ^25^, while BF possibly contain fragments of organic particles ^8^ ^,^ ^19^. However, this did not compromise the biological effects of bone healing in our present study. Furthermore, the various sources of the bovine graft, whether from cortical or medullary, may have affected the results of this study. BF is manufactured from the cortical bone might explain the more intensified TRAP-positive cells involved in reabsorption of this biomaterial and its slower bone formation.

Further research is warranted to evaluate both biomaterials including molecular analysis, to shed light on the strengths and limitations of BO and BF. Regarding the limitations of this study, it should be stated that this is a rodent animal model which only partially simulate the clinical condition. Clinical studies using a split mouth design with a large sample should be conducted to reflect the clinical scenario in humans. Ultimately, these future studies can promote insights in the way for more effective bone grafting interventions, improving patient outcomes and quality of life.

In conclusion, even with the higher expression of proteins related to bone formation, there was no difference in new bone formation at 45 days when both types of anorganic bovine xenogenous grafts were evaluated.
